# Unveiling the veil of lactate in tumor-associated macrophages: a successful strategy for immunometabolic therapy

**DOI:** 10.3389/fimmu.2023.1208870

**Published:** 2023-07-26

**Authors:** Hongxia Tao, Xuansheng Zhong, Anqi Zeng, Linjiang Song

**Affiliations:** ^1^ School of Medical and Life Sciences, Chengdu University of Traditional Chinese Medicine, Chengdu, Sichuan, China; ^2^ Clinical Medicine Department, Bengbu Medical College, Bengbu, China; ^3^ Institute of Translational Pharmacology and Clinical Application, Sichuan Academy of Chinese Medical Science, Chengdu, Sichuan, China

**Keywords:** lactate, TAMs, immune escape, immunometabolism, targeted drug delivery

## Abstract

Lactate, traditionally regarded as a metabolic waste product at the terminal of the glycolysis process, has recently been found to have multifaceted functional roles in metabolism and beyond. A metabolic reprogramming phenomenon commonly seen in tumor cells, known as the “Warburg effect,” sees high levels of aerobic glycolysis result in an excessive production of lactate. This lactate serves as a substrate that sustains not only the survival of cancer cells but also immune cells. However, it also inhibits the function of tumor-associated macrophages (TAMs), a group of innate immune cells ubiquitously present in solid tumors, thereby facilitating the immune evasion of malignant tumor cells. Characterized by their high plasticity, TAMs are generally divided into the pro-inflammatory M1 phenotype and the pro-tumour M2 phenotype. Through a process of ‘education’ by lactate, TAMs tend to adopt an immunosuppressive phenotype and collaborate with tumor cells to promote angiogenesis. Additionally, there is growing evidence linking metabolic reprogramming with epigenetic modifications, suggesting the participation of histone modification in diverse cellular events within the tumor microenvironment (TME). In this review, we delve into recent discoveries concerning lactate metabolism in tumors, with a particular focus on the impact of lactate on the function of TAMs. We aim to consolidate the molecular mechanisms underlying lactate-induced TAM polarization and angiogenesis and explore the lactate-mediated crosstalk between TAMs and tumor cells. Finally, we also touch upon the latest progress in immunometabolic therapies and drug delivery strategies targeting glycolysis and lactate production, offering new perspectives for future therapeutic approaches.

## 1 Introduction

Macrophages are typically the most common immune cell type and are ubiquitous in body tissues. Acting as a bridge between natural and acquired immunity, macrophages not only directly engulf and eliminate pathogens or dead cells, but also act as antigen-presenting cells (APCs) to process and deliver antigens to trigger anti-tumour effects (1, 2). Tumour-associated macrophages (TAMs) are abundantly infiltrated in tumor microenvironment (TME), accounting for more than 50% of solid tumours, and are functionally plastic and heterogeneous (3, 4). In normal tissues, most macrophages undergo a coordinated functional transition between M1 and M2 phenotypes, contributing to the promotion of acute inflammation and tissue repair, respectively (5). Although in the early stages of cancer, TAMs exhibit a pro-inflammatory M1 phenotype to perform anti-tumour effects (6). However, they were ultimately transformed to an immunosuppressive and pro-angiogenic M2 phenotype under the education of TME, thereby facilitating tumour growth and evasion of immune surveillance (7).

In solid tumours, hypoxic zones are prevalent where cells use anaerobic glycolysis to convert pyruvate to lactate under low oxygen conditions (8). In 1923, Otto Heinrich Warburg discovered the phenomenon of aerobic glycolysis in tumour cells under conditions of sufficient oxygen, termed the “Warburg effect” (9). Three main advantages of the “Warburg effect” are known, including a rapid increase in adenosine triphosphate (ATP) levels, the provision of biosynthetic intermediates and the prevention of cellular oxidative stress (10, 11). In addition, this effect has also been found to be present in rapidly proliferating immune cells (12). Previous studies have focused on intermediate metabolites of the tricarboxylic acid (TCA) cycle, such as citric, succinic and fumaric acids (13). Lactate was initially considered to be a by-product of anaerobic glycolysis and was misidentified as a waste product (14). However, lactate has received much attention in recent years. Under physiological conditions (pH 7.2), lactate is present as sodium lactate (15). Whereas in TME at low pH, lactate exists as lactic acid (15). In 2014, lactate was first demonstrated to induce TAM polarization (16). The significant role of lactate in tumor immune escape has also been investigated, but the mechanisms underlying the activation of the pro-angiogenic phenotype of TAMS by lactate remain to be explored, and studies for a comprehensive insight into lactate-targeted immunotherapy are limited.

This review concentrates on the unique role of lactate-activated TAMs in aberrant angiogenesis and immune modulation, with an highlight on recent advances in lactate-targeted drugs and delivery strategies. A detailed description of lactate-mediated interactions between immune cells provides a theoretical basis for a better understanding of the non-metabolic functions of lactate, as well as a potentially valuable avenue for immunometabolic therapies.

## 2 Phenotypic and immunomodulatory effect of TAM in tumors

### 2.1 Phenotypic diversity and metabolic patterns of macrophages

**Figure 1:**
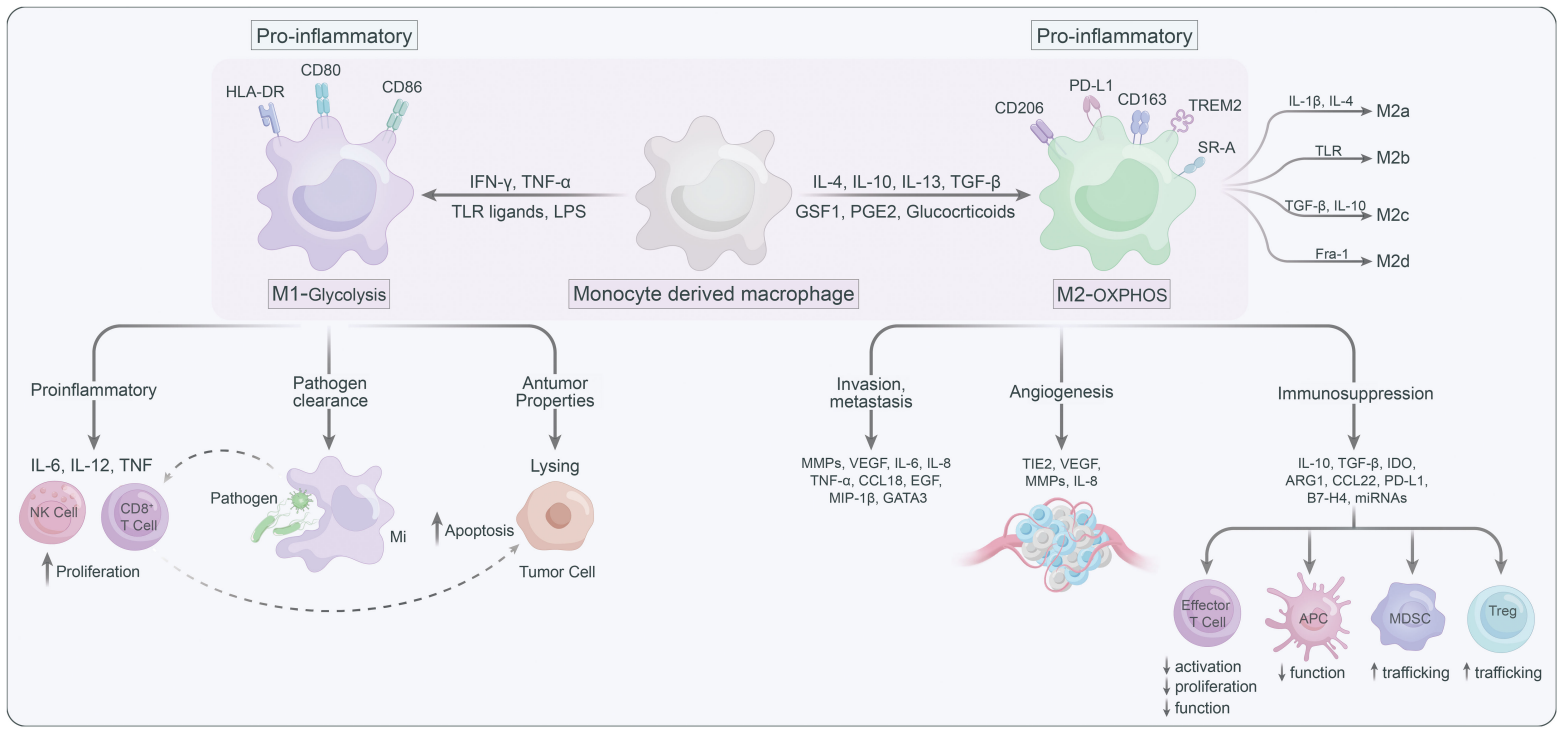
The characteristics of M1 versus M2 macrophages.

Macrophages are highly plastic and can be polarized into different states, generally summarized as the classically activated M1 macrophages (anti-tumoral phenotype) and the alternatively activated M2 macrophages (pro-tumoral phenotype) (17) (**Figure 1**). Several signaling molecules, such as PI3K/Akt–ERK signaling (18), STAT3 (19), HIF1α (20), and STAT6 (21) are involved in macrophage M2-polarization. Generally, metabolic signals and the prototypical polarizing signals like IFN-γ and LPS leading to TAMs to express the M1 phenotype, or IL-4 and IL-10 can convert TAMs into the M2 state (22). Notably, M2 macrophages could further be classified into M2a, M2b, M2c and M2d according to their functional state (23). TAMs are thought to predominantly belong to M2d, which can be stimulated by lactate (24, 25). In addition to phenotypic diversity, TAMs also exhibit heterogeneity in their metabolic patterns, which contribute to their functional differences. M1 macrophages, which mostly by relying on glycolysis and pentose phosphate pathway (PPP), are known for their pro-inflammatory roles (26). Furthermore, the pro-inflammatory TAMs can promote proliferation and recruitment of cytotoxic immune cells such as CD8^+^ T and NK cells to support the tumor-suppressive functions of the pro inflammatory (M1-type) TAMs (27). While M2 macrophages are generally featured with enhanced oxidative phosphorylation (OXPHOS) and fatty acid oxidation (FAO), can facilitate tumor progression by immune suppression and promotion of cancer cell migration/invasion, angiogenesis, and metastasis (28). In light of this, targeting metabolic pathways has emerged as an attractive strategy for modulating the phenotypes of TAMs and their subsequent immune responses. By altering the metabolic preferences of TAMs, one could potentially shift their functions, providing a novel approach for the treatment of various diseases including cancer (29–31).

### 2.2 TAM-mediated tumor immune escape in the TME

Blocking inhibitory immune checkpoint proteins like PD-1, PD-L1, and CTLA-4 with monoclonal antibodies has revolutionized cancer treatment by counteracting cancer cells’ evasion of immune responses and restoring T cell function (32). Unfortunately, a significant challenge in current immunological-checkpoint blockade therapies is that over 60% of patients do not experience any benefits due to resistance observed in certain tumors (33). The concept of “immune editing” elucidates the mechanisms behind the continued growth of tumors despite the presence of a fully operational host immune system (34). During this process, tumor cell division can give rise to reduced immunogenicity, allowing the tumor to evade immune detection by employing immunosuppressive effects or by losing the expression of target antigens (35). This immune evasion is likely to be influenced by factors such as the existing infiltration of immune cells within the tumor during treatment, the composition of the tumor stroma, and the mechanisms of immune resistance (30).

Macrophages as essential tissue-resident antigen-presenting cells, modulating immune responses by transitioning from a pro-inflammatory phenotype to an immunosuppressive phenotype that facilitates tumor growth (36, 37). This non-inflammatory phenotype is distinguished by transcriptional downregulation of iNOS, TNF, IL-12, and Toll-like receptor (TLR) signaling pathway components, alongside transcriptional upregulation of arginase 1, C-type lectin CLEC10A, and IL-10 (38, 39). In the polyoma middle T antigen model of breast cancer, CD4+ T cell–derived IL-4 is thought to be responsible for the functional shift towards a non-inflammatory phenotype (40). The non-inflammatory polarization of macrophages promotes the expression of PD-L1 in monocytes, leading to the suppression of cytotoxic T cell responses (41). Consequently, tumors manipulate macrophages to establish a microenvironment that facilitates tumor growth by inducing angiogenesis, enhancing tumor cell migration, and promoting invasion (42). This non-inflammatory macrophage polarization as a key aspect of tumor immunoevasion highlights its central role in shaping the TME.

## 3 Glycolysis, lactate, and tumor microenvironment

### 3.1 Generation of lactate in the tumour microenvironment

The Warburg effect, a hallmark of tumor cells, manifests as aerobic glycolysis, wherein the primary metabolic fate of glucose is its conversion into lactate, despite the presence of oxygen (43, 44). Both cancer cells and stromal cells release significant quantities of lactate into the TME due to the presence of the Warburg effect and reverse Warburg effect (45).. Prominently found within the TME, alongside immune system elements like macrophages and lymphocytes, are cells comprising blood vessels, fibroblasts, myofibroblasts, mesenchymal stem cells, adipocytes, and the extracellular matrix (ECM), with TAMs stand out as a prominent constituent (25). In addition to tumor-derived lactate, tumor cells employ miR-375 to reprogram TAMs into lactate producers, thereby fulfilling the energy demands of tumor cells (46).

While tumor cells were once thought to be the primary generators of lactate and protons in the TME for several decades, emerging evidence now reveals that immune and stromal populations within tumors play a crucial role in substantial “lactification” and acidification contributions (47–49). TGF-β signaling from cancer cells induces the Warburg effect in cancer-associated fibroblasts, leading to lactate secretion (50, 51). This lactate can be absorbed by neighboring tumor cells through the “reverse Warburg effect” (50). Immune cells also undergo a metabolic transition, resembling Warburg metabolism, to support their functions (52). The PI3K/Akt/mTORC1 pathway plays a critical role in promoting aerobic glycolysis in both immune cells and tumor cells (29). Studies using [18F]FDG-PET have shown increased glucose metabolism in immune cells, with activated T-lymphocytes exhibiting higher glucose uptake in immunocompetent mice (53–55). TAMs, monocytes, and neutrophils exhibit greater glycolytic dependency compared to dendritic cells (56). Tumor-infiltrating T cells have limited glycolytic activity due to glucose competition from highly glycolytic tumor cells (49, 57, 58). The lactate levels in the TME are closely linked to immune cell infiltration and metabolism. Furthermore, co-culture experiments have revealed that tumor cells can induce lactate production in TAMs via the miR-375-LDH-B axis (46).

### 3.2 Impact of lactate on macrophages in normal and non-cancer pathologies

Lactate, often perceived merely as a byproduct of cellular metabolism, actually serves as a significant signaling molecule that plays a crucial role in regulating normal physiology and pathology beyond the scope of cancer (59, 60). Under normal circumstances, systemic lactate concentrations are stringently maintained at approximately 1-2 mM. However, these concentrations can escalate to an exceptional 40 mM within the TME, potentially impacting cellular function (61). Studies have indicated that tumor cells from both humans and mice excrete lactate *in vitro*, with substantial amounts of lactate being associated with tumor metastasis and unfavorable clinical prognosis (16, 62, 63).

Lactate is involved in various processes such as inflammation, wound healing, and immune response modulation, with macrophages being key players in these processes (64). During inflammation, lactate act as an energy source for cells, ensuring their function and survival (65). Moreover, lactate is not just a fuel but can also regulate immune cell behavior, specifically macrophages (66). For instance, lactate can influence macrophage polarization, which is an important aspect of the innate immune response (67). Beyond the inflammatory response, lactate has a critical role in wound healing. High concentrations of lactate can promote the migration of macrophages which subsequently transition from a pro-inflammatory M1 phenotype to a pro-healing M2 phenotype (66). As such, lactate serves as a mediator for wound healing by orchestrating the dynamic function of macrophages in the process (66). Further, lactate also plays a significant role in sepsis, a severe systemic inflammatory response syndrome (68). Studies have indicated that lactate produced during sepsis can impair the function of macrophages and other immune cells, contributing to immune dysfunction, a hallmark of sepsis (69, 70). Lactate also influences the process of antigen presentation by macrophages, affecting the host’s adaptive immune response (68). Thus, targeting lactate metabolism in macrophages could be a potential therapeutic strategy for sepsis management.

In addition to the aforementioned pathologies, lactate has also been implicated in various other conditions such as neurodegenerative diseases, diabetes, and myocardial infarction (71–73). The common thread in all these pathological conditions is the role of lactate in the modulation of macrophage function and the associated inflammatory responses. It is noteworthy that the lactate concentration in the microenvironment dictates the impact on macrophage function. Under physiological conditions, lactate concentration is relatively low, and thus its impact on macrophages is minimal (74). However, under pathological conditions, the dramatic increase in lactate concentrations can significantly impair macrophage function (75). Understanding the exact mechanisms through which lactate impacts macrophage function in non-cancer pathologies could yield essential insights into the design of novel therapeutics for these diseases.

## 4 Function of lactate in TAM polarization and angiogenesis

**Figure 2:**
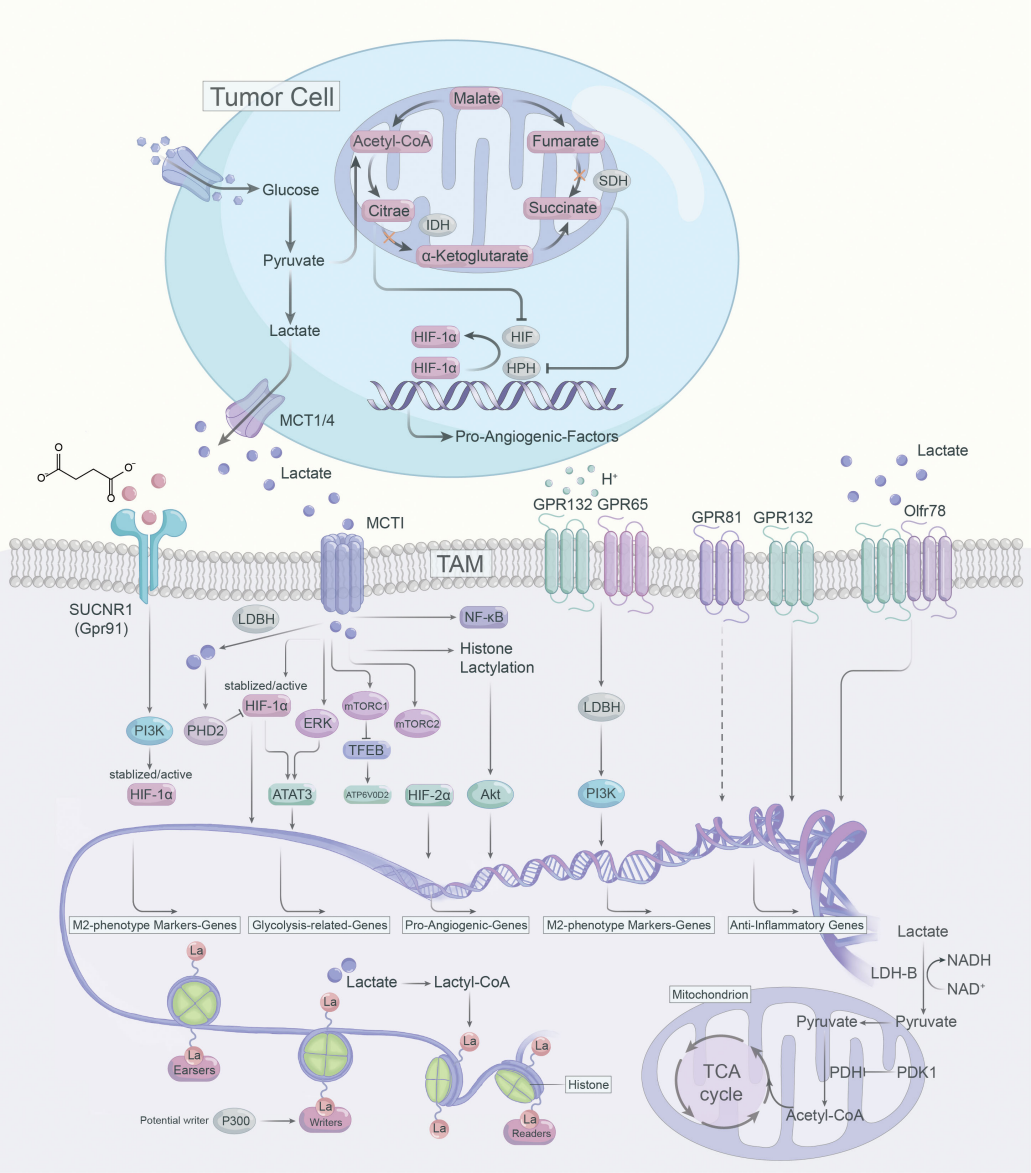
Lactate-mediated signaling pathways in the polarization of TAMs.

**Figure 3:**
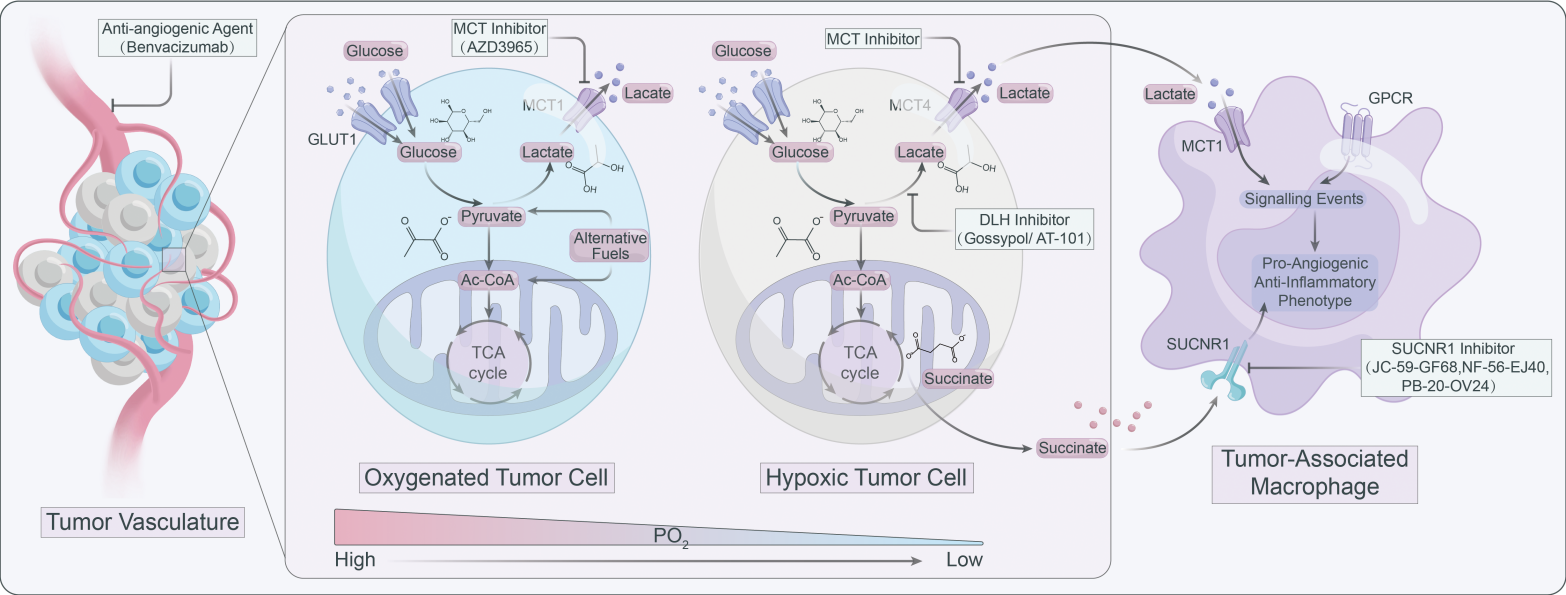
Targeting lactate signaling is a promising strategy to counteract resistance against angiogenic therapy.

Lactate operates as an active signaling molecule that exerts control over the polarization and function of TAMs via receptor-mediated signaling pathways (**Figure 2**). Exposure of TAMs to lactate elicits a pro-angiogenic phenotype, implicating their crucial involvement in the dysregulation of angiogenic therapies. Hence, targeting lactate production, shuttling, and/or signaling may offer a promising approach to counteract resistance against angiogenic therapies (**Figure 3**).

### 4.1 Lactate-monocarboxylate transporters signaling

Lactate build-up can lead to “self-poisoning” and impair the glycolytic process (76). Excessive accumulation of lactate lowers cytoplasmic pH, leading to inhibition of alkaline pH-dependent phosphofructokinase (PFK) and LDH activity, which results in attenuated glycolysis (77). In TME, lactate can induce anti-inflammatory and pro-angiogenic ATMs phenotypes via multiple pathways. For instance, lactate can be transported bidirectionally via MCTs belonging to the SLC16A family, as MCTs are precise regulators in modulating lactate export and import (78, 79). MCT1 and MCT2 can promote lactate efflux in a substrate-dependent concentration gradient, whereas MCT3 and MCT4 are efficient lactate exporters that promote lactate efflux from highly glycolytic cells (79). However, MCT1 and MCT4 are highly expressed in numerous tumors and are linked to adverse prognosis (80). The expression of MCT1 and MCT4 positively correlated with CD163^+^ TAMs in human breast cancer and oral squamous cell carcinoma, respectively (81, 82). MCT1 enables lactate influx that promotes glycolysis and M2 phenotypic polarization of TAMS, while knockdown of MCT4 blocks the glycolytic process by decreasing LDH-A expression (83). LDH-A-mediated lactate production increases the NAD/NADH ratio, which is critical for CD40 signaling, and CD40-induced lactate production by fine-tuning the NAD/NADH ratio for M1 macrophage polarization rather than M2 polarization (84).

#### 4.1.1 HIF-α

Contrary to LDH-A, LDH-B preferentially oxidizes lactate to pyruvate while reducing NAD to NADH, and pyruvate competes with α-ketoglutarate to suppress prolyl hydroxylase 2 (PHD2) activity and further inhibit HIF-1α (85). Further, HIF-1α can enhance glycolytic activity via upregulating PDK1 expression and blocking the import of lactate-derived pyruvate into mitochondria for the TCA cycle (86). By stabilizing HIF-1α, lactate could enhance VEGF and Arg1 expression and induces M2-like polarization of TAMs, and VEGF and Arg1 support tumor growth via inducing neovascularization and providing substrates for cancer cell proliferation, respectively (16).

In tumor cells and activated ATMs, intracellular citrate and succinate inhibit HIF-α inhibitory factors (87, 88). Citrate, which accumulates due to the downregulation of isocitrate dehydrogenase (IDH), directly inhibits HIF-α asparagine hydroxylase (FIH) (87). As for succinate, which gets as a result of SDH inhibition, inhibits HIF prolyl hydroxylases (HPHs) (87, 88). Both result in the stabilization and activation of HIF-α and hence the transcription of pro-angiogenic VEGF (87, 88). In breast cancer, sodium/glucose cotransporter 1 (SGLT1) overexpression drives high glycolysis in tumor cells, and secreted lactate promotes polarization of M2 TAMs via activating the HIF-1α/STAT3 signaling, promoting the polarization of M2 phenotype TAMs, thus creating a vicious cycle between breast cancer cells and TAMs (89). In addition, lactate-mediated activation of the ERK/STAT3 signaling pathway was determined to perform a crucial role in the pro-angiogenic M2-like polarization of ATMs and subsequent tumor neovascularization (25). Similarly, in cervical cancer, lactate contributes to the M2 phenotype of TAMs by promoting HIF-1α expression and downregulating nuclear factor kappa-B (NF-κB) phosphorylation in TAMs (89).

#### 4.1.2 mTOR

There are insufficient studies on mTOR in ATM. The TSC2-mTOR pathway has been identified as a critical regulator of monocyte differentiation to M2-like TAM (90). TSC2 knockdown activates mTOR, converting macrophages to an angiogenic-promoting M2-like phenotype and increasing the release of IL-10 (90). Conversely, rapamycin inhibits mTOR, leading to the differentiation of monocytes into M1-like TAM (90). Consistently, knockdown of REDD1, an inhibitor of mTOR, impedes glycolysis in TAM and inhibits excessive tumor angiogenesis and metastasis (91). Lactate activates mTORC1 via MCTs and represses TFEB, while suppressing the expression of ATP6V0d2 (a subunit of V-ATPase), a target gene of TFEB (92). However, ATP6V0d2 promotes HIF-2α lysosomal degradation, blocking ATP6V0d2 induces HIF-2α activation, which promotes VEGF expression and enhances the pro-angiogenic effect of TAMs (92). Furthermore, in pituitary adenomas, lactate can contribute to tumor invasion via activating mTORC2/Akt signaling (93).

#### 4.1.3 Histone lactylation

Lactate has been considered as a byproduct of sugar metabolism, especially in glycolysis, but in 2019, Zhao et al. first identified and demonstrated that lactate can promote lactonization modifications on histone lysine residues, which in turn directly regulates transcriptional expression of genes, terming it histone lysine lactylation (Kla) (94). TME is rich in lactate and the accumulation of lactate could induce histone Kla in TAMs. The authors used LPS+IFN-γ to induce polarization of Bone marrow-derived macrophages (BMDMs) toward M1 phenotype macrophages and found not only a progressive increase in intracellular lactate levels over 24 hours, but also an increasing level of histone Kla (94). It was also found that the expression of the M2-like gene (Arg1) increased continuously with the increase of the Kla level in M1 macrophages. The experimental results suggest that as histone Kla increases, macrophages polarize toward the M2 phenotype, and even macrophages that are already M1 phenotype repolarize toward the M2 phenotype (94). Notably, lactate, an epigenetic regulatory molecule, can induce the expression of Arg1 and other homologous genes involved in wound healing via histone lysine Kla, promoting the function of M2 phenotype TAMs (94). In addition, the acetyltransferase enzyme p300 is involved in the process of histone Kla (95). Lactyl-CoA produced from lactate contributes a lactyl (La) group via p300 to the lysine tail of histones, activating pro-wound healing genes and leading to an M2-like phenotype (95). However, which enzymes generate the intermediate molecule lactyl-CoA, from which La is derived, and which enzymes deposit (writers), remove (erasers) or recognize and interpret (readers) histone lactylation remain to be identified (96).

### 4.2 Lactate-G protein–coupled receptors signaling

#### 4.2.1 Gpr132

Except MCTs, the G protein-coupled receptor 132 (Gpr132, or G2A) and Gpr65 (or TDAG8) are functional lactate receptor both highly expressed on the surface of macrophages (30, 97, 98). Gpr132 serve as a stress-inducible, seven-pass transmembrane receptor whose activation blocks the cell cycle and modulates proliferation and immunity (99, 100). It was found that cancer cell-derived lactate activated Gpr132 on ATMs to promote the M2 phenotype, and that lactate-Gpr132 axis-activated ATMs enhanced tumor cell metastasis, thus forming a positive feedback loop (97). Gpr132 activation produces inositol triphosphate (IP3) via Gq activation, and increases intracellular calcium concentration (99). Notably, although Gpr132 is also a proton receptor, GPR132 deletion specifically affects calcium mobilization triggered by lactate, rather than by hydrochloric acid (HCI) (97). This suggests that Gpr132 not only acts as a functional receptor for lactate, but that lactate is a key signal for activating Gpr132 (97). Lower Gpr132 expression is associated with better survival in breast cancer patients, so targeting the lactate-Gpr132 axis may help to abrogate the M2 phenotypic polarization of TAMs and lung metastasis of breast cancer (97). Further, activation of the lactate-G2A-PPARγ-axis can mediate the M2 polarization of ATMs and promote breast cancer metastasis (97, 101).

#### 4.2.2 Gpr65

Bohn et al. found that in malignant melanoma, lactate promoted anti-inflammatory M2-like TAM polarization through activation of GPR65 (30). Moreover, Lailler reported that in glioblastoma (GBM), the expression of the lactate receptor GPR65 was highest at the mRNA level (102). Interestingly, lactate may be dispensable for the pro-angiogenic TAMs phenotype and anti-inflammatory function mediated by GPCRs (30, 103). Acidic TME was demonstrated to directly activate proton-sensing Gpr65 and Gpr132 on TAMs, independent of lactate, leading to cyclic AMP (cAMP) accumulation, which induces the expression of cAMP early inhibitory factor (ICER) and M2 phenotypic markers (104, 105). In addition, ICER acts as a potent inhibitor of gene transcription, blocking the expression of pro-inflammatory factors such as TNF-α (30, 105).

#### 4.2.3 Olfr78

Odorant receptors (ORs) are the largest subfamily of GPCRs (106). 2021, Vadevoo et al. explored the role of an OR termed Olfr78 in TAM (107). Olfr78 recognizes lactate and mediates lactate-induced M2 polarization of macrophages (107). Furthermore, Olfr78 forms a heterodimer with Gpr132, which promotes M2 macrophage polarization, and subsequently promotes tumor progression and metastasis (107). The combined effect of Olfr78 and Gpr132 enhanced Olfr78 expression and lactate-responsive activity, and conversely, Olfr78 or Gpr132 deficiency reduced lactate-mediated M2 polarization of ATMs (107). However, the potential molecular mechanisms downstream of lactate-Olfr78/Gpr132 signaling in TAMs remain to be explored.

#### 4.2.4 Gpr81

Gpr81 is the most intensively studied lactate receptor in mediating intracellular signaling (108). The expression of Gpr81 is diverse depending on the cell type and tissue microenvironment. For instance, Gpr81 is highly expressed on adipocytes and has been reported to flourish on macrophages in the intestine and lung (108, 109). In addition, DCs in the TME express high levels of Gpr81 (110). Highly expressed Gpr81 is emerging as a critical regulator of tumor growth and metastasis, lactate can modulate Gpr81 expression in lung tumor cells (111). In the TME, lactate-Gpr81 signaling plays an essential role in facilitating PD-L1 expression and chemotherapy resistance (112, 113). Additionally, the expression of MCT1 and MCT4 in tumor cells is regulated by lactate-GPR81 signaling (112). Further, lactate-Gpr81 signaling has been reported to be regulated by the PPARγ in adipocytes and the snail 3/STAT3 pathways in tumor cells pathways (111, 114, 115). In a preclinical model of mouse breast cancer, lactate activates Gpr81 in plasmacytoid dendritic cells (pDCs), inducing intracellular calcium mobilization and inhibiting IFNα production, thus augmenting immunosuppression in TME (116). In monocytes and macrophages, lactate-Gpr81 signaling activation suppresses inflammatory responses by limiting the activation of the β-arrestin/inflammasome pathway, mediates the histoprotective effect (117). Besides, lactate-Gpr81 signaling exerts a role in the suppression of macrophage proinflammatory cytokine production in response to LPS stimulation via inhibition of yes-associated protein (YAP) and NF-κB activation (118). In contrast to its anti-inflammatory role, lactate boosts LPS-stimulated TLR4 activation and NF-κB-dependent inflammatory gene expression in macrophages via monocarboxylate transporters and MD-2 up-regulation (119). Taken together, the above studies suggest that the lactate-Gpr81 signaling axis has a regulatory role in macrophage immune function, and it may also play an essential role in the pro-tumor function of TAMs, but remains to be determined.

## 5 Lactate mediates the communication between TAMs and tumor cells

### 5.1 Lactate as a metabolic fuel for tumour cells

Constitutive glucose uptake, a characteristic of cancer cells, supplies the energy and biosynthetic components necessary for their rapid proliferation (120). Glucose carbon in cultured cancer cells undergoes glycolysis to generate pyruvate, which is then converted to lactate by lactate dehydrogenase (LDH) and secreted (121). This disposal of carbon as lactate fulfills multiple roles, including the NADH-dependent recycling of NAD+ during glycolysis by LDH and the elimination of protons through monocarboxylate transporters, maintaining intracellular pH homeostasis and acidifying the extracellular space (29). Furthermore, tumors utilize lactate as a fuel source, thereby expanding its metabolic functions within the context of cancer (122). Brandon et al. demonstrated the preference of human non-small cell lung cancer (NSCLC) for lactate over glucose as a primary fuel source to sustain tumor metabolism *in vivo* by fueling the tricarboxylic acid (TCA) cycle (29). Moreover, the application of sodium oxalate in hepatocellular carcinoma (HCC) effectively suppressed lactate dehydrogenase and aerobic glycolysis, resulting in a notable reduction in lactate concentrations, ultimately compromising the viability and proliferation of tumorigenic HCC cells (123). In line with this, a study by Sheng et al. revealed that lactate as the primary contributor to circulating TCA intermediates in fasted mice with genetically engineered lung and pancreatic cancer tumors, even in the presence of glucose, highlighting its significant role in the metabolic turnover, particularly in the context of various tissues and tumors (124). As evidenced by *in vivo* isotope tracing, melanoma xenografts with a higher propensity for metastasis showed greater incorporation of lactate into TCA metabolites via MCT1-facilitated absorption compared to their counterparts with lower metastatic potential (125). In essence, the primary use of lactate in the TCA cycle to produce ATP may separate energy production from aerobic glycolysis, possibly enabling glucose to fuel other cell proliferation and metastasis processes (124, 125). Taken together, lactate is a key metabolic fuel for mouse and human tumor cells *in vivo*, hinting at a link between lactate uptake and metastasis, though more research is needed.

### 5.2 Lactate supports immunosuppression of TAMs

Previously deemed a glycolysis waste product, lactate is now recognized as a critical mediator in tumor-immune cell interactions, facilitating immune evasion (126–128). Elevated lactic acid levels can create an acidic cellular environment, suppressing immune cells like macrophages, T cells and NK cells, thereby fostering tumor proliferation and metastasis (16, 97, 129, 130). TAMs constitute over half of the immune cells in the tumor microenvironment, M1-TAMs enable antigen presentation and immune factor activation, promoting anti-tumor responses (131). In contrast, M2-TAMs dampen inflammation, evade tumor immune surveillance, and stimulate tumor growth and metastasis (132). Lactate can instigate the MCT-HIF1α pathway, prompting macrophages to polarize towards M2, thereby augmenting the regulatory mechanism of macrophage polarization (133). This transition results in a decrease in IL-12 and an increase in IL-10 expression in M2-TAMs, fostering tumor development (134). Lactate can also deter HIF2α degradation by activating mTORC1 in macrophages, further bolstering tumor growth (92). Furthermore, lactate can impede YAP and nuclear factor-kB activation via a GPR81-mediated signal, curtailing pro-inflammatory cytokine production in macrophages, thus inhibiting their pro-inflammatory response to LPS stimulation (118). Modulating lactate levels can facilitate the shift of macrophages from M1 to M2 and enhance PD-L1 expression, aiding in tumor immune evasion (135). Additionally, research suggests that reducing tumor lactate levels can deter M2 macrophage polarization, inhibiting CCL17 secretion and curtailing pituitary adenoma invasion (93). A comprehensive grasp of lactate’s effects on TAMs is key, not only for countering the evasion tactics of tumor cells against anti-tumor immune responses but also for forecasting how lactate metabolism could shape targeted therapies in cancer treatment.

## 6 Targeting lactate and glycolysis for immunometabolic therapy

**Figure 4:**
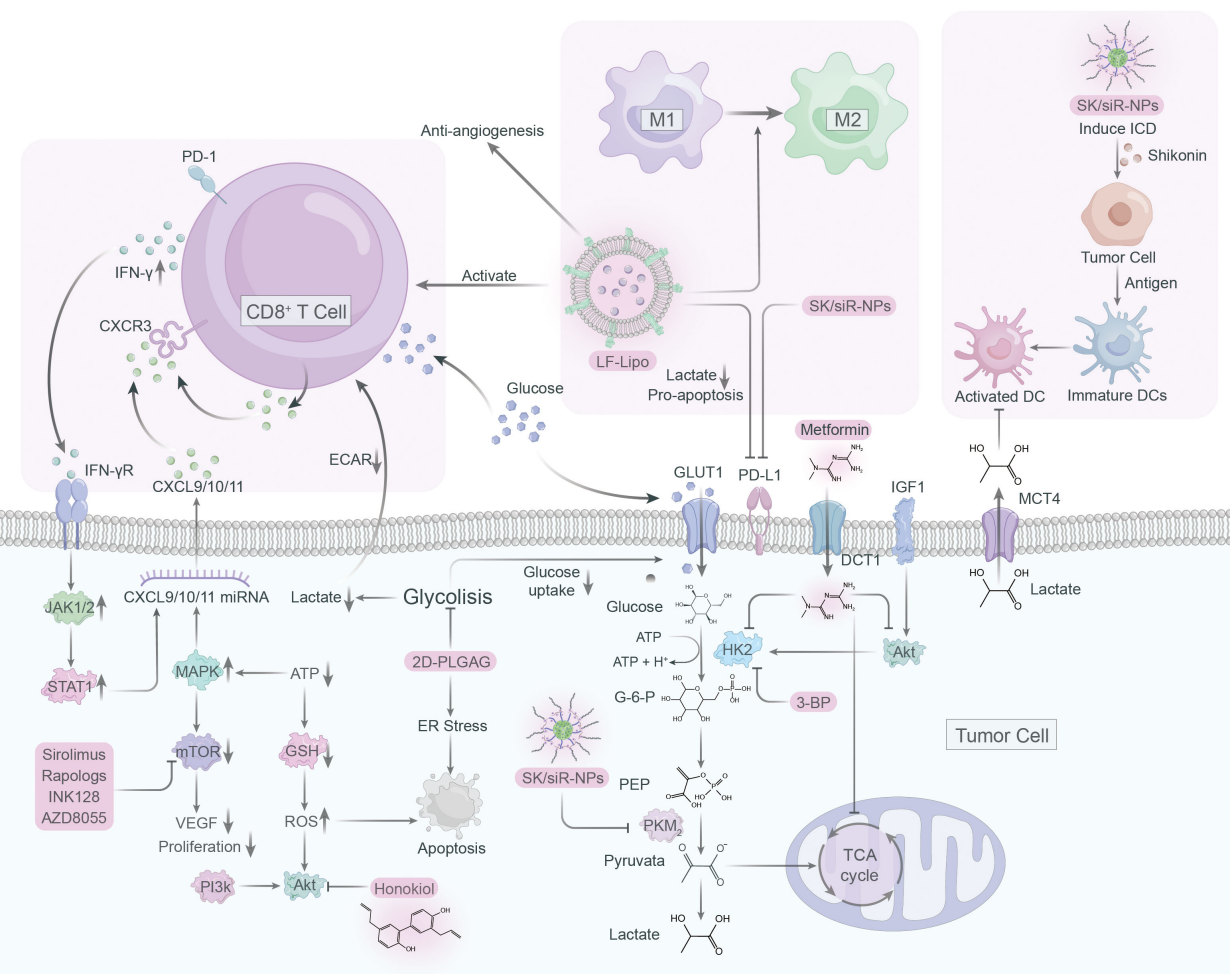
Immunometabolic therapy targeting lactate and glycolysis.

Although only a few drugs have been studied in clinical trials targeting proteins associated with lactate in macrophages, we present an overview of immunometabolic therapies targeting glycolysis and lactate (**Figure 4**).

### 6.1 Targeting pyruvate kinase M2

PKM2, an essential glycolytic enzyme, is frequently overexpressed in a variety of solid tumors, playing a critical role in tumorigenesis (136–138). As such, it has become an attractive target for anti-cancer drug development. Shikonin and its analogs alkannin have shown promising results as PKM2-specific inhibitors, effectively reducing the energy supply of tumor cells by interfering with glycolysis (139). Remarkably, these compounds demonstrated a similar effect on drug-sensitive and resistant tumor cells, suggesting their clinical potential (139). However, the limited water solubility of shikonin has hindered its clinical application (140, 141). To solve this issue and improve tumor immunotherapy, a mannosylated lactoferrin nanoparticulate system (Man-LF NPs) has been developed for the co-delivery of shikonin and JQ1, an effective inhibitor of PD-L1 (142). Man-LF NPs could significantly inhibit PD-L1 and reduce lactate production in tumor cells, thus enhancing the therapeutic efficacy of immunotherapy (142). In addition, a versatile nanoparticle codelivering shikonin and PD-L1 knockdown siRNA (SK/siR-NPs) could effectively suppress PD-L1 and reduce lactate production by inhibiting PKM2 (143). SK/siR-NPs has been reported to have significant potential in tumor immunotherapy manifested by induction of immunogenic cell death (ICD), enhanced response of effector T cells, repolarization of TAMs and DC maturation (143).

### 6.2 Targeting mTOR

The mTOR pathway plays a vital role in tumorigenesis by regulating metabolism and promoting tumor growth, making it a promising target for cancer therapy (144). However, specific inhibitors such as rapamycin have limited bioavailability and solubility in water, rendering them less effective (145). To overcome this, a liposome system was developed to co-deliver rapamycin and regorafenib, an anti-angiogenic drug, effectively educing the lactate production and promoting antitumor immune responses (146). Natural compound honokiol has been shown to inhibit the PI3K/mTOR pathway and reduce immune resistance in tumors, but its limited penetrance through the blood-brain barrier has made it difficult to use in treating gliomas (147). A brain-targeted liposomal delivery system was developed by using a peptide that binds to α7 nicotinic acetylcholine receptors (nAChRs), which are overexpressed on glioma cells and TAMs (148). By employing this system, honokiol and disulfiram/copper were effectively delivered to the tumor site, leading to the inhibition of glucose metabolism, lactate production, M2 to M1 TAM repolarization, triggering of tumor autophagy, and promoting antitumor immunity (148, 149).

### 6.3 Targeting hexokinase

Hexokinase is a vital enzyme in glycolysis that that contributes to the development of tumor cells by priming glucose to glucose-6-phosphate, with hexokinase 2 (HK2) being the most active isozyme (150). Though 2-deoxy-D-glucose (2-DG) is a competitive inhibitor of glycolysis that can preferentially kill tumor cells, its toxicity raises concerns about its application in cancer therapy (151, 152). To address this, 2DG-loaded PLGA nanoparticles (2DG-PLGA-NPs) were developed, which effectively promote tumor cell apoptosis, enhance IFN-γ production in CD8^+^ T cells, and resist anti-PD-1 resistance when combined with sorafenib or anti-PD1 (153). Metformin, a typical diabetic drug, has potential antitumor properties by inhibiting glycolysis and glycogen synthesis (154). Metformin can reduce HK2 activity and impairs glycolysis, as well as indirectly inhibit HK2 by inhibiting IGF1-induced AKT phosphorylation (155). When combined with BPTES nanoparticles, metformin showed an enhanced effect on pancreatic tumor reduction (156).

### 6.4 Targeting epigenetics

Epigenetic readers such as BET proteins, notably BRD4, have been shown to upregulate PD-L1 expression, promoting tumor growth (157, 158). JQ1, a BRD4 inhibitor, can suppress PD-L1 expression and inhibit lactate production (159). Liposomal targeting codelivery of JQ1 and ecognizedt, a histone deacetylase inhibitor, can improve treatment efficacy via epigenetic regulation, with tumor cells and TAM highly expressing the lipoprotein receptor-associated protein 1 (LRP-1) receptor (159). By using lactoferrin-modified targeted liposomal system (LF-Lipo) to co-deliver ecognizedt and JQ1, binding with LRP-1 receptors on tumor cells and TAMs can reduce lactate production, repolarize M2 phenotype TAMs, and enhance CD8^+^ T cell antitumor and anti-angiogenesis responses (159).

### 6.5 Targeting glycolysis

Targeting lactate metabolism through blocking glycolysis holds great potential as an efficacious strategy for cancer therapy. 3-BP, a halogenated analog of pyruvate, has emerged as a promising anti-tumor agent due to its selective inhibition of critical glycolysis enzymes including HK2, GAPDH, and 3-PGK, thus reducing ATP production and causing cancer cell death (160, 161). However, clinical applications of 3-BP are limited due to poor pharmacokinetics and systemic toxicity. To overcome these challenges, nanoparticle-based drug delivery technologies, such as liposomes and nanoparticles, have been developed as a targeted drug delivery solution to enhance drug efficacy and reduce toxicity (162). These groundbreaking advancements provide an exceptionally promising avenue for enhancing drug delivery, as they offer finely-tuned specificity and bolstered efficacy.

## 7 Conclusion

Lactate in TME is essential and was previously considered to be a metabolic waste product. We summarize recent evidence that lactate has a broad role in tumour immunity. Kla is a novel post-translational modification (PTM) with lactate as a substrate. The discovery of kla and its effect on macrophages helped to unravel the mystery of the Warburg effect. Although kla was shown to be a consequence of reparative gene expression in macrophages rather than a cause (163), inhibitors of key proteins in lactate metabolism show great promise in preclinical studies. **Table 1** summarizes recent advances in drugs targeting lactate metabolism, but these drugs are still worthy of continued exploration in human trials.

**Table 1:** Pharmaceutical drugs targeting lactate metabolism in tumors.

Target	Inhibitors	Clinical trials
LDH-A	GNE-140, NHI, Galloflavin, FX-11, Stiripentol, Nifuroxazide, Honokiol, Oxamate, Sirtinol, Quiflapon	Phase II/III (Pancreatic cancer, Small cell lung cancer, Non-small cell lung cancer, Esophageal cancer, Ocular melanoma, Intracranial malignant tumor, Rhabdomyosarcoma)
LDH-B	Quinoline 3‐sulfonamides, NHI	None
GLUT1	WZB117, STF-31 KRH3955, BAY-876 STF-118804, STF 31, Rapamycin, Fucoxanthin, Quercetin	None
Hexokinase	3-BrPA, 2-DG, DMJ, Lonidamine, Sorafenib, WZB-117, STF-31, 7ACC2 STF-31, PFK-158, KGP94, CG-5	Phase II (Ovarian cancer, Pancreatic cancer, Prostate cancer)
MCT1	AZD3965, AR-C155858, GW-1100, SR13800, AZB1109, Chrysin, Quercetin, Pterostilbene, DIDS, SLC-0111, 7ACC2	Phase II (Non-small cell lung cancer, Melanoma) Phase I/II (Head and neck cancer, Melanoma, Colorectal cancer)
MCT4	BAY-8002, Pterostilbene, CHC, Naringenin, Arctigenin, AZD3965, BAY-8002, CB-839, DIDS	Phase III (Renal cell carcinoma)
HIF-1α	Echinomycin, PX-478, 2ME, YC-1, Digoxin, DCA, Resveratrol, LW6, TEI-6720, Curcumin, 2-DG	Phase II (Glioblastoma, Renal carcinoma, Kaposi’s sarcoma)
PKM2	Shikonin, DASA-10, TEPP-46, Sulfasalazine, ML265, DASA-58, MEDI3039, GSK-2837808A, Thyroid hormone, EGCG	Preclinical studies
mTOR	Rapamycin, Everolimus, Temsirolimus, AZD8055, NVP-BEZ-235, PP242, Gedatolisib, OSI-027, AZD2014, MLN0128	Phase II (Breast cancer, Renal cell carcinoma)
Histone deacetylases (HDACs)	Sodium butyrate, Trichostatin A, Vorinostat, Valproic acid, Mocetinostat	Preclinical studies
Bromodomain and extra-terminal (BET) proteins	I-BET151, JQ1, OTX015, TEN-010, ABBV-075	Preclinical studies
Glycolysis	3-BP	Preclinical studies

NHI, N‐hydroxyindole; FX11, 3-dihydroxy-6-methyl-7-(phenylmethyl)-4-propylnaphthalene-1-carboxylic acid; 3-BrPA, 3-bromopyruvate; 2-DG, 2-deoxy-D-glucose; DMJ, deoxymannojirimycin; DIDS, 4,4′-diisothiocyanatostilbene-2,2′-disulfonic acid disodium salt hydrate; 7ACC2, 7-(N-benzyl-N-methylamino)-2-oxo-2H-chromene-3-carboxylic acid; CHC, α-cyano-4-hydroxycinnamate; EGCG, epigallocatechin-3-gallate; 3-BP, 3-Brormopyruvate.

Over the past decade, cancer immunotherapy has been ecognized as one of the most promising therapeutic strategies. A large amount of research is currently focusing on PD-1/PD-L1-targeted drugs to restore or enhance isolated components of the immune system, but only a small number of individuals have had appreciable results. Metabolic checkpoints have attracted significant attention as promising therapeutic targets to enhance the anti-cancer immune response, and therefore exploring strategies to target metabolic pathways is a necessary and feasible direction of research.

The metabolism of tumor cells is highly plastic, overlapping with that of normal cells and differing *in vitro* and *in vivo* environments, leading to significant challenges in the development of pharmaceuticals that currently target cancer metabolism. The development of drug delivery technologies and physical strategies for integrated therapy may address these limitations, for example, nanotechnology-based targeted delivery and phototherapy.

In conclusion, the understanding of the role of lactate in tumor metabolic reprogramming and tumor immunity is still only the tip of the iceberg up to now, and further exploration will bring about interesting discoveries.

## Author contributions

LS and AZ were involved in the conception of the study. HT and XZ were involved in writing the article. LS and AZ critically revised the manuscript. All authors contributed to the article and approved the submitted version.

## Glossary

APCs: antigen-presenting cells
TAMs: tumor-associated macrophages
TME: tumor microenvironment
ATP: adenosine triphosphate
TCA: tricarboxylic acid
PPP: pentose phosphate pathway
OXPHOS: oxidative phosphorylation
FAO: fatty acid oxidation
TLR: Toll-like receptor
ECM: extracellular matrix
MCTs: monocarboxylate transporters
PFK: phosphofructokinase
PHD2: prolyl hydroxylase 2
IDH: isocitrate dehydrogenase
HPHs: HIF prolyl hydroxylases
SGLT1: sodium/glucose cotransporter 1
NF-κB: nuclear factor kappa-B
Kla: histone lysine lactylation
BMDMs: bone marrow-derived macrophages
GPCRs: G protein–coupled receptors
La: lactyl
IP3: inositol triphosphate
HCI: hydrochloric acid
GBM: glioblastoma
cAMP: cyclic AMP
Ors: odorant receptors
pDCs: plasmacytoid dendritic cells
YAP: yes-associated protein
LDH: lactate dehydrogenase
non-small cell lung cancer: NSCLC
TCA: tricarboxylic acid
HCC: hepatocellular carcinoma
PKM2: pyruvate kinase M2
Man-LF NPs: mannosylated lactoferrin nanoparticulate system
SK/siR-NPs: nanoparticle codelivering shikonin and PD-L1 knockdown siRNA
ICD: immunogenic cell death
nAChRs: α7 nicotinic acetylcholine receptors
HK2: hexokinase 2
2-DG: 2-deoxy-D-glucose
2DG-PLGA-NPs: 2DG-loaded PLGA nanoparticles
LRP-1: lipoprotein receptor-associated protein 1
LF-Lipo: lactoferrin-modified targeted liposomal system

## References

[1] Sun J Muz B Alhallak K Markovic M Gurley S Wang Z. Targeting CD47 as a Novel Immunotherapy for Multiple Myeloma. Cancers (Basel) (2020) 12(2):305. doi: 10.3390/cancers12020305 PMC7072283 32012878

[2] He K Jia S Lou Y Liu P Xu LX. Cryo-thermal therapy induces macrophage polarization for durable anti-tumor immunity. Cell Death Dis (2019) 10(3):216. doi: 10.1038/s41419-019-1459-7 30833570 PMC6399266

[3] Zhang Y Tang J Wang C Zhang Q Zeng A Song L. Autophagy-related lncRNAs in tumor progression and drug resistance: A double-edged sword. Genes Dis (2023). doi: 10.1016/j.gendis.2023.04.015 PMC10425854 37588204

[4] He R He Y Du R Liu C Chen Zeng A Song L. Revisiting of TAMs in tumor immune microenvironment: Insight from NF-κB signaling pathway. BioMed Pharmacother (2023) 165:115090. doi: 10.1016/j.biopha.2023.115090 37390708

[5] Garabuczi χÉ Tarban N Fige χÉ Patsalos A Halász L Szendi-Szatmári T. Nur77 and PPARγ regulate transcription and polarization in distinct subsets of M2-like reparative macrophages during regenerative inflammation. Front Immunol (2023) 14:1139204. doi: 10.3389/fimmu.2023.1139204 36936920 PMC10020500

[6] He Z Zhang S. Tumor-Associated Macrophages and Their Functional Transformation in the Hypoxic Tumor Microenvironment. Front Immunol (2021) 12:741305. doi: 10.3389/fimmu.2021.741305 34603327 PMC8481680

[7] Yeung OW Lo CM Ling CC Qi X Geng W Li CX. Alternatively activated (M2) macrophages promote tumour growth and invasiveness in hepatocellular carcinoma. J Hepatol (2015) 62(3):607–16. doi: 10.1016/j.jhep.2014.10.029 25450711

[8] Leung E Cairns RA Chaudary N Vellanki RN Kalliomaki T Moriyama EH. Metabolic targeting of HIF-dependent glycolysis reduces lactate, increases oxygen consumption and enhances response to high-dose single-fraction radiotherapy in hypoxic solid tumors. BMC Cancer (2017) 17(1):418. doi: 10.1186/s12885-017-3402-6 28619042 PMC5473006

[9] Warburg O Wind F Negelein E. THE METABOLISM OF TUMORS IN THE BODY. J Gen Physiol (1927) 8(6):519–30. doi: 10.1085/jgp.8.6.519 PMC2140820 19872213

[10] Hayes C Donohoe CL Davern M Donlon NE. The oncogenic and clinical implications of lactate induced immunosuppression in the tumour microenvironment. Cancer Lett (2021) 500:75–86. doi: 10.1016/j.canlet.2020.12.021 33347908

[11] Pavlova NN Thompson CB. The Emerging Hallmarks of Cancer Metabolism. Cell Metab (2016) 23(1):27–47. doi: 10.1016/j.cmet.2015.12.006 26771115 PMC4715268

[12] Xu K Yin N Peng M Stamatiades EG Chhangawala S Shyu A. Glycolytic ATP fuels phosphoinositide 3-kinase signaling to support effector T helper 17 cell responses. Immunity (2021) 54(5):976–987.e977. doi: 10.1016/j.immuni.2021.04.008 33979589 PMC8130647

[13] Yang S Zhao J Cui X Zhan Q Yi K Wang Q. TCA-phospholipid-glycolysis targeted triple therapy effectively suppresses ATP production and tumor growth in glioblastoma. Theranostics (2022) 12(16):7032–50. doi: 10.7150/thno.74197 PMC9576613 36276638

[14] Haas R Cucchi D Smith J Pucino V Macdougall CE Mauro C. Intermediates of Metabolism: From Bystanders to Signalling Molecules. Trends Biochem Sci (2016) 41(5):460–71. doi: 10.1016/j.tibs.2016.02.003 26935843

[15] Haas R Smith J Rocher-Ros V Nadkarni S Montero-Melendez T D'Acquisto F. Lactate Regulates Metabolic and Pro-inflammatory Circuits in Control of T Cell Migration and Effector Functions. PloS Biol (2015) 13(7):e1002202. doi: 10.1371/journal.pbio.1002202 26181372 PMC4504715

[16] Colegio OR Chu NQ Szabo AL Chu T Rhebergen AM Jairam V. Functional polarization of tumour-associated macrophages by tumour-derived lactic acid. Nature (2014) 513(7519):559–63. doi: 10.1038/nature13490 PMC4301845 25043024

[17] Oyarce C Vizcaino-Castro A Chen S Boerma A Daemen T. Re-polarization of immunosuppressive macrophages to tumor-cytotoxic macrophages by repurposed metabolic drugs. Oncoimmunology (2021) 10(1):1898753. doi: 10.1080/2162402X.2021.1898753 33796407 PMC7971325

[18] Zhang W Xu W Xiong S. Macrophage differentiation and polarization via phosphatidylinositol 3-kinase/Akt-ERK signaling pathway conferred by serum amyloid P component. J Immunol (2011) 187(4):1764–77. doi: 10.4049/jimmunol.1002315 21753147

[19] Sun L Chen B Jiang R Li J Wang B. Resveratrol inhibits lung cancer growth by suppressing M2-like polarization of tumor associated macrophages. Cell Immunol (2017) 311:86–93. doi: 10.1016/j.cellimm.2016.11.002 27825563

[20] Raggi F Pelassa S Pierobon D Penco F Gattorno M Novelli F. Regulation of Human Macrophage M1-M2 Polarization Balance by Hypoxia and the Triggering Receptor Expressed on Myeloid Cells-1. Front Immunol (2017) 8:1097. doi: 10.3389/fimmu.2017.01097 28936211 PMC5594076

[21] Rahman K Vengrenyuk Y Ramsey SA Vila NR Girgis NM Liu J. Inflammatory Ly6Chi monocytes and their conversion to M2 macrophages drive atherosclerosis regression. J Clin Invest (2017) 127(8):2904–15. doi: 10.1172/JCI75005 PMC5531402 28650342

[22] Arora S Dev K Agarwal B Das P Syed MA. Macrophages: Their role, activation and polarization in pulmonary diseases. Immunobiology (2018) 223(4-5):383–96. doi: 10.1016/j.imbio.2017.11.001 PMC7114886 29146235

[23] Smith RJ Jr. Nasiri B Kann J Yergeau D Bard JE Swartz DD. Endothelialization of arterial vascular grafts by circulating monocytes. Nat Commun (2020) 11(1):1622. doi: 10.1038/s41467-020-15361-2 32238801 PMC7113268

[24] Motta JM Rumjanek VM Mantovani A Locati M. Tumor-Released Products Promote Bone Marrow-Derived Macrophage Survival and Proliferation. Biomedicines (2021) 9(10):1387. doi: 10.3390/biomedicines9101387 PMC8533124 34680504

[25] Mu X Shi W Xu Y Xu C Zhao T Geng B. Tumor-derived lactate induces M2 macrophage polarization via the activation of the ERK/STAT3 signaling pathway in breast cancer. Cell Cycle (2018) 17(4):428–38. doi: 10.1080/15384101.2018.1444305 PMC5927648 29468929

[26] Morgan PK Huynh K Pernes G Miotto PM Mellett NA Giles C. Macrophage polarization state affects lipid composition and the channeling of exogenous fatty acids into endogenous lipid pools. J Biol Chem (2021) 297(6):101341. doi: 10.1016/j.jbc.2021.101341 34695418 PMC8604758

[27] Jassar AS Suzuki E Kapoor V Sun J Silverberg MB Cheung L. Activation of tumor-associated macrophages by the vascular disrupting agent 5,6-dimethylxanthenone-4-acetic acid induces an effective CD8+ T-cell-mediated antitumor immune response in murine models of lung cancer and mesothelioma. Cancer Res (2005) 65(24):11752–61. doi: 10.1158/0008-5472.CAN-05-1658 16357188

[28] Batista-Gonzalez A Vidal R Criollo A Carreño LJ. New Insights on the Role of Lipid Metabolism in the Metabolic Reprogramming of Macrophages. Front Immunol (2019) 10:2993. doi: 10.3389/fimmu.2019.02993 31998297 PMC6966486

[29] Faubert B Li KY Cai L Hensley CT Kim J Zacharias LG. Lactate Metabolism in Human Lung Tumors. Cell (2017) 171(2):358–371.e359. doi: 10.1016/j.cell.2017.09.019 28985563 PMC5684706

[30] Bohn T Rapp S Luther N Klein M Bruehl TJ Kojima N. Tumor immunoevasion via acidosis-dependent induction of regulatory tumor-associated macrophages. Nat Immunol (2018) 19(12):1319–29. doi: 10.1038/s41590-018-0226-8 30397348

[31] Chaudagar K Hieromnimon HM Khurana R Labadie B Hirz T Mei S. Reversal of Lactate and PD-1-mediated Macrophage Immunosuppression Controls Growth of PTEN/p53-deficient Prostate Cancer. Clin Cancer Res (2023) 29(10):1952–68. doi: 10.1158/1078-0432.CCR-22-3350 PMC10192075 36862086

[32] Zak KM Grudnik P Magiera K Dömling A Dubin G Holak TA. Structural Biology of the Immune Checkpoint Receptor PD-1 and Its Ligands PD-L1/PD-L2. Structure (2017) 25(8):1163–74. doi: 10.1016/j.str.2017.06.011 28768162

[33] Wolchok JD Kluger H Callahan MK Postow MA Rizvi NA Lesokhin AM. Nivolumab plus ipilimumab in advanced melanoma. N Engl J Med (2013) 369(2):122–33. doi: 10.1056/NEJMoa1302369 PMC5698004 23724867

[34] Dunn GP Bruce AT Ikeda H Old LJ Schreiber RD. Cancer immunoediting: from immunosurveillance to tumor escape. Nat Immunol (2002) 3(11):991–8. doi: 10.1038/ni1102-991 12407406

[35] Vinay DS Ryan EP Pawelec G Talib WH Stagg J Elkord E. Immune evasion in cancer: Mechanistic basis and therapeutic strategies. Semin Cancer Biol (2015) 35 Suppl:S185–s198. doi: 10.1016/j.semcancer.2015.03.004 25818339

[36] Saccani A Schioppa T Porta C Biswas SK Nebuloni M Vago L. p50 nuclear factor-kappaB overexpression in tumor-associated macrophages inhibits M1 inflammatory responses and antitumor resistance. Cancer Res (2006) 66(23):11432–40. doi: 10.1158/0008-5472.CAN-06-1867 17145890

[37] Pollard JW. Trophic macrophages in development and disease. Nat Rev Immunol (2009) 9(4):259–70. doi: 10.1038/nri2528 PMC3648866 19282852

[38] Biswas SK Gangi L Paul S Schioppa T Saccani A Sironi M. A distinct and unique transcriptional program expressed by tumor-associated macrophages (defective NF-kappaB and enhanced IRF-3/STAT1 activation). Blood (2006) 107(5):2112–22. doi: 10.1182/blood-2005-01-0428 16269622

[39] Ojalvo LS King W Cox D Pollard JW. High-density gene expression analysis of tumor-associated macrophages from mouse mammary tumors. Am J Pathol (2009) 174(3):1048–64. doi: 10.2353/ajpath.2009.080676 PMC2665764 19218341

[40] DeNardo DG Barreto JB Andreu P Vasquez L Tawfik D Kolhatkar N. CD4(+) T cells regulate pulmonary metastasis of mammary carcinomas by enhancing protumor properties of macrophages. Cancer Cell (2009) 16(2):91–102. doi: 10.1016/j.ccr.2009.06.018 19647220 PMC2778576

[41] Kuang DM Zhao Q Peng C Xu J Zhang JP Wu C. Activated monocytes in peritumoral stroma of hepatocellular carcinoma foster immune privilege and disease progression through PD-L1. J Exp Med (2009) 206(6):1327–37. doi: 10.1084/jem.20082173 PMC2715058 19451266

[42] Qian BZ Pollard JW. Macrophage diversity enhances tumor progression and metastasis. Cell (2010) 141(1):39–51. doi: 10.1016/j.cell.2010.03.014 20371344 PMC4994190

[43] Koppenol WH Bounds PL Dang CV. Otto Warburg's contributions to current concepts of cancer metabolism. Nat Rev Cancer (2011) 11(5):325–37. doi: 10.1038/nrc3038 21508971

[44] Cassim S Vučetić M Ždralević M Pouyssegur J. Warburg and Beyond: The Power of Mitochondrial Metabolism to Collaborate or Replace Fermentative Glycolysis in Cancer. Cancers (Basel) (2020) 12(5):1119. doi: 10.3390/cancers12051119 PMC7281550 32365833

[45] Tekade RK Sun X. The Warburg effect and glucose-derived cancer theranostics. Drug Discovery Today (2017) 22(11):1637–53. doi: 10.1016/j.drudis.2017.08.003 28843632

[46] Frank AC Raue R Fuhrmann DC Sirait-Fischer E Reuse C Weigert A. Lactate dehydrogenase B regulates macrophage metabolism in the tumor microenvironment. Theranostics (2021) 11(15):7570–88. doi: 10.7150/thno.58380 PMC8210612 34158867

[47] Jeong H Kim S Hong BJ Lee CJ Kim YE Bok S. Tumor-Associated Macrophages Enhance Tumor Hypoxia and Aerobic Glycolysis. Cancer Res (2019) 79(4):795–806. doi: 10.1158/0008-5472.CAN-18-2545 30610087

[48] Shangguan C Gan G Zhang J Wu J Miao Y Zhang M. Cancer-associated fibroblasts enhance tumor (18)F-FDG uptake and contribute to the intratumor heterogeneity of PET-CT. Theranostics (2018) 8(5):1376–88. doi: 10.7150/thno.22717 PMC5835943 29507627

[49] Reinfeld BI Madden MZ Wolf MM Chytil A Bader JE Patterson AR. Cell-programmed nutrient partitioning in the tumour microenvironment. Nature (2021) 593(7858):282–8. doi: 10.1038/s41586-021-03442-1 PMC8122068 33828302

[50] Pavlides S Whitaker-Menezes D Castello-Cros R Flomenberg N Witkiewicz AK Frank PG. The reverse Warburg effect: aerobic glycolysis in cancer associated fibroblasts and the tumor stroma. Cell Cycle (2009) 8(23):3984–4001. doi: 10.4161/cc.8.23.10238 19923890

[51] Guido C Whitaker-Menezes D Capparelli C Balliet R Lin Z Pestell RG. Metabolic reprogramming of cancer-associated fibroblasts by TGF-β drives tumor growth: connecting TGF-β signaling with "Warburg-like" cancer metabolism and L-lactate production. Cell Cycle (2012) 11(16):3019–35. doi: 10.4161/cc.21384 PMC3442913 22874531

[52] Kornberg MD Bhargava P Kim PM Putluri V Snowman AM Putluri N. Dimethyl fumarate targets GAPDH and aerobic glycolysis to modulate immunity. Science (2018) 360(6387):449–53. doi: 10.1126/science.aan4665 PMC5924419 29599194

[53] Pektor S Bausbacher N Otto G Lawaczeck L Grabbe S Schreckenberger M. Toll like receptor mediated immune stimulation can be visualized in *vivo* by [(18)F]FDG-PET. Nucl Med Biol (2016) 43(11):651–60. doi: 10.1016/j.nucmedbio.2016.07.004 27552488

[54] Yamada S Kubota K Kubota R Ido T Tamahashi N. High accumulation of fluorine-18-fluorodeoxyglucose in turpentine-induced inflammatory tissue. J Nucl Med (1995) 36(7):1301–6. 7790960

[55] Kubota R Yamada S Kubota K Ishiwata K Tamahashi N Ido T. Intratumoral distribution of fluorine-18-fluorodeoxyglucose in *vivo*: high accumulation in macrophages and granulation tissues studied by microautoradiography. J Nucl Med (1992) 33(11):1972–80. doi: 10.2967/jnumed.120.251744a 1432158

[56] Argüello RJ Combes AJ Char R Gigan JP Baaziz AI Bousiquot E. SCENITH: A Flow Cytometry-Based Method to Functionally Profile Energy Metabolism with Single-Cell Resolution. Cell Metab (2020) 32(6):1063–1075.e1067. doi: 10.1016/j.cmet.2020.11.007 33264598 PMC8407169

[57] Chang CH Qiu J O'Sullivan D Buck MD Noguchi T Curtis JD. Metabolic Competition in the Tumor Microenvironment Is a Driver of Cancer Progression. Cell (2015) 162(6):1229–41. doi: 10.1016/j.cell.2015.08.016 PMC4864363 26321679

[58] DePeaux K Delgoffe GM. Metabolic barriers to cancer immunotherapy. Nat Rev Immunol (2021) 21(12):785–97. doi: 10.1038/s41577-021-00541-y PMC8553800 33927375

[59] Semenza GL. Targeting HIF-1 for cancer therapy. Nat Rev Cancer (2003) 3(10):721–32. doi: 10.1038/nrc1187 13130303

[60] Sonveaux P Copetti T De Saedeleer CJ Végran F Verrax J Kennedy KM. Targeting the lactate transporter MCT1 in endothelial cells inhibits lactate-induced HIF-1 activation and tumor angiogenesis. PloS One (2012) 7(3):e33418. doi: 10.1371/journal.pone.0033418 22428047 PMC3302812

[61] Walenta S Wetterling M Lehrke M Schwickert G Sundfør K Rofstad EK. High lactate levels predict likelihood of metastases, tumor recurrence, and restricted patient survival in human cervical cancers. Cancer Res (2000) 60(4):916–21. 10706105

[62] Fischer K Hoffmann P Voelkl S Meidenbauer N Ammer J Edinger M. Inhibitory effect of tumor cell-derived lactic acid on human T cells. Blood (2007) 109(9):3812–9. doi: 10.1182/blood-2006-07-035972 17255361

[63] Wang Z Embaye KS Yang Q Qin L Zhang C Liu L. Establishment and validation of a prognostic signature for lung adenocarcinoma based on metabolism-related genes. Cancer Cell Int (2021) 21(1):219. doi: 10.1186/s12935-021-01915-x 33858449 PMC8050921

[64] Pucino V Nefla M Gauthier V Alsaleh G Clayton SA Marshall J. Differential effect of lactate on synovial fibroblast and macrophage effector functions. Front Immunol (2023) 14:1183825. doi: 10.3389/fimmu.2023.1183825 37304267 PMC10251493

[65] Ye L Jiang Y Zhang M. Crosstalk between glucose metabolism, lactate production and immune response modulation. Cytokine Growth Factor Rev (2022) 68:81–92. doi: 10.1016/j.cytogfr.2022.11.001 36376165

[66] Zhou HC Xin-Yan Y Yu WW Liang XQ Du XY Liu ZC. Lactic acid in macrophage polarization: The significant role in inflammation and cancer. Int Rev Immunol (2022) 41(1):4–18. doi: 10.1080/08830185.2021.1955876 34304685

[67] Zhou HC Yu WW Yan XY Liang XQ Ma XF Long JP. Lactate-driven macrophage polarization in the inflammatory microenvironment alleviates intestinal inflammation. Front Immunol (2022) 13:1013686. doi: 10.3389/fimmu.2022.1013686 36330516 PMC9623299

[68] Yang K Fan M Wang X Xu J Wang Y Tu F. Lactate promotes macrophage HMGB1 lactylation, acetylation, and exosomal release in polymicrobial sepsis. Cell Death Differ (2022) 29(1):133–46. doi: 10.1038/s41418-021-00841-9 PMC8738735 34363018

[69] Broder G Weil MH. Excess lactate: an index of reversibility of shock in human patients. Science (1964) 143(3613):1457–9. doi: 10.1126/science.143.3613.1457 14107454

[70] Singer M Deutschman CS Seymour CW Shankar-Hari M Annane D Bauer M. The Third International Consensus Definitions for Sepsis and Septic Shock (Sepsis-3). Jama (2016) 315(8):801–10. doi: 10.1001/jama.2016.0287 PMC4968574 26903338

[71] Lambert V Hansen S Schoumacher M Lecomte J Leenders J Hubert P. Pyruvate dehydrogenase kinase/lactate axis: a therapeutic target for neovascular age-related macular degeneration identified by metabolomics. J Mol Med (Berl) (2020) 98(12):1737–51. doi: 10.1007/s00109-020-01994-9 33079232

[72] Cai H Wang X Zhang Z Chen J Wang F Wang L. Moderate l-lactate administration suppresses adipose tissue macrophage M1 polarization to alleviate obesity-associated insulin resistance. J Biol Chem (2022) 298(4):101768. doi: 10.1016/j.jbc.2022.101768 35218776 PMC8941214

[73] Zhang J Huang F Chen L Li G Lei W Zhao J. Sodium Lactate Accelerates M2 Macrophage Polarization and Improves Cardiac Function after Myocardial Infarction in Mice. Cardiovasc Ther (2021) 2021:5530541. doi: 10.1155/2021/5530541 34194542 PMC8203388

[74] Crowther M Brown NJ Bishop ET Lewis CE. Microenvironmental influence on macrophage regulation of angiogenesis in wounds and malignant tumors. J Leukoc Biol (2001) 70(4):478–90. doi: 10.1189/jlb.70.4.478 11590184

[75] Swallow CJ Grinstein S Rotstein OD. Lipopolysaccharide impairs macrophage cytoplasmic pH regulation under conditions simulating the inflammatory microenvironment. J Leukoc Biol (1992) 52(4):395–9. doi: 10.1002/jlb.52.4.395 1402389

[76] Caruso JP Koch BJ Benson PD Varughese E Monterey MD Lee AE. pH, Lactate, and Hypoxia: Reciprocity in Regulating High-Affinity Monocarboxylate Transporter Expression in Glioblastoma. Neoplasia (2017) 19(2):121–34. doi: 10.1016/j.neo.2016.12.011 PMC5238458 28092823

[77] Pérez-Tomás R Pérez-Guillén I. Lactate in the Tumor Microenvironment: An Essential Molecule in Cancer Progression and Treatment. Cancers (Basel) (2020) 12(11):3244. doi: 10.3390/cancers12113244 PMC7693872 33153193

[78] Beloueche-Babari M Casals Galobart T Delgado-Goni T Wantuch S Parkes HG Tandy D. Monocarboxylate transporter 1 blockade with AZD3965 inhibits lipid biosynthesis and increases tumour immune cell infiltration. Br J Cancer (2020) 122(6):895–903. doi: 10.1038/s41416-019-0717-x 31937921 PMC7078321

[79] Bosshart PD Kalbermatter D Bonetti S Fotiadis D. Mechanistic basis of L-lactate transport in the SLC16 solute carrier family. Nat Commun (2019) 10(1):2649. doi: 10.1038/s41467-019-10566-6 31201333 PMC6573034

[80] Kobayashi M Narumi K Furugen A Iseki K. Transport function, regulation, and biology of human monocarboxylate transporter 1 (hMCT1) and 4 (hMCT4). Pharmacol Ther (2021) 226:107862. doi: 10.1016/j.pharmthera.2021.107862 33894276

[81] Li B Yang Q Li Z Xu Z Sun S Wu Q. Expression of Monocarboxylate Transporter 1 in Immunosuppressive Macrophages Is Associated With the Poor Prognosis in Breast Cancer. Front Oncol (2020) 10:574787. doi: 10.3389/fonc.2020.574787 33178603 PMC7596686

[82] Bisetto S Whitaker-Menezes D Wilski NA Tuluc M Curry J Zhan T. Monocarboxylate Transporter 4 (MCT4) Knockout Mice Have Attenuated 4NQO Induced Carcinogenesis; A Role for MCT4 in Driving Oral Squamous Cell Cancer. Front Oncol (2018) 8:324. doi: 10.3389/fonc.2018.00324 30211114 PMC6120975

[83] Kaushik DK Bhattacharya A Mirzaei R Rawji KS Ahn Y Rho JM. Enhanced glycolytic metabolism supports transmigration of brain-infiltrating macrophages in multiple sclerosis. J Clin Invest (2019) 129(8):3277–92. doi: 10.1172/JCI124012 PMC6668690 31112527

[84] Liu PS Chen YT Li X Hsueh PC Tzeng SF Chen H. CD40 signal rewires fatty acid and glutamine metabolism for stimulating macrophage anti-tumorigenic functions. Nat Immunol (2023) 24(3):452–62. doi: 10.1038/s41590-023-01430-3 PMC9977680 36823405

[85] Zhao Y Zhao B Wang X Guan G Xin Y Sun YD. Macrophage transcriptome modification induced by hypoxia and lactate. Exp Ther Med (2019) 18(6):4811–9. doi: 10.3892/etm.2019.8164 PMC6878900 31798707

[86] Baltazar F Afonso J Costa M Granja S. Lactate Beyond a Waste Metabolite: Metabolic Affairs and Signaling in Malignancy. Front Oncol (2020) 10:231. doi: 10.3389/fonc.2020.00231 32257942 PMC7093491

[87] Koivunen P Hirsilä M Remes AM Hassinen IE Kivirikko KI Myllyharju J. Inhibition of hypoxia-inducible factor (HIF) hydroxylases by citric acid cycle intermediates: possible links between cell metabolism and stabilization of HIF. J Biol Chem (2007) 282(7):4524–32. doi: 10.1074/jbc.M610415200 17182618

[88] Selak MA Armour SM MacKenzie ED Boulahbel H Watson DG Mansfield KD. Succinate links TCA cycle dysfunction to oncogenesis by inhibiting HIF-alpha prolyl hydroxylase. Cancer Cell (2005) 7(1):77–85. doi: 10.1016/j.ccr.2004.11.022 15652751

[89] Niu X Ma J Li J Gu Y Yin L Wang Y. Sodium/glucose cotransporter 1-dependent metabolic alterations induce tamoxifen resistance in breast cancer by promoting macrophage M2 polarization. Cell Death Dis (2021) 12(6):509. doi: 10.1038/s41419-021-03781-x 34006822 PMC8131586

[90] Chen W Ma T Shen XN Xia XF Xu GD Bai XL. Macrophage-induced tumor angiogenesis is regulated by the TSC2-mTOR pathway. Cancer Res (2012) 72(6):1363–72. doi: 10.1158/0008-5472.CAN-11-2684 22287548

[91] Wenes M Shang M Di Matteo M Goveia J Martín-Pérez R Serneels J. Macrophage Metabolism Controls Tumor Blood Vessel Morphogenesis and Metastasis. Cell Metab (2016) 24(5):701–15. doi: 10.1016/j.cmet.2016.09.008 27773694

[92] Liu N Luo J Kuang D Xu S Duan Y Xia Y. Lactate inhibits ATP6V0d2 expression in tumor-associated macrophages to promote HIF-2α-mediated tumor progression. J Clin Invest (2019) 129(2):631–46. doi: 10.1172/JCI123027 PMC6355226 30431439

[93] Zhang A Xu Y Xu H Ren J Meng T Ni Y. Lactate-induced M2 polarization of tumor-associated macrophages promotes the invasion of pituitary adenoma by secreting CCL17. Theranostics (2021) 11(8):3839–52. doi: 10.7150/thno.53749 PMC7914368 33664865

[94] Zhang D Tang Z Huang H Zhou G Cui C Weng Y. Metabolic regulation of gene expression by histone lactylation. Nature (2019) 574(7779):575–80. doi: 10.1038/s41586-019-1678-1 PMC6818755 31645732

[95] Jin M Cao W Chen B Xiong M Cao G. Tumor-Derived Lactate Creates a Favorable Niche for Tumor via Supplying Energy Source for Tumor and Modulating the Tumor Microenvironment. Front Cell Dev Biol (2022) 10:808859. doi: 10.3389/fcell.2022.808859 35646923 PMC9136137

[96] Chen AN Luo Y Yang YH Fu JT Geng XM Shi JP. Lactylation, a Novel Metabolic Reprogramming Code: Current Status and Prospects. Front Immunol (2021) 12:688910. doi: 10.3389/fimmu.2021.688910 34177945 PMC8222712

[97] Chen P Zuo H Xiong H Kolar MJ Chu Q Saghatelian A. Gpr132 sensing of lactate mediates tumor-macrophage interplay to promote breast cancer metastasis. Proc Natl Acad Sci USA (2017) 114(3):580–5. doi: 10.1073/pnas.1614035114 PMC5255630 28049847

[98] Bolick DT Skaflen MD Johnson LE Kwon SC Howatt D Daugherty A. G2A deficiency in mice promotes macrophage activation and atherosclerosis. Circ Res (2009) 104(3):318–27. doi: 10.1161/CIRCRESAHA.108.181131 PMC2716803 19106413

[99] Murakami N Yokomizo T Okuno T Shimizu T. G2A is a proton-sensing G-protein-coupled receptor antagonized by lysophosphatidylcholine. J Biol Chem (2004) 279(41):42484–91. doi: 10.1074/jbc.M406561200 15280385

[100] Radu CG Yang LV Riedinger M Au M Witte ON. T cell chemotaxis to lysophosphatidylcholine through the G2A receptor. Proc Natl Acad Sci U.S.A. (2004) 101(1):245–50. doi: 10.1073/pnas.2536801100 PMC314170 14681556

[101] Cheng WY Huynh H Chen P Peña-Llopis S Wan Y. Macrophage PPARγ inhibits Gpr132 to mediate the anti-tumor effects of rosiglitazone. Elife (2016) 5:e18501. doi: 10.7554/eLife.18501 PMC5047746 27692066

[102] Lailler C Louandre C Morisse MC Lhossein T Godin C Lottin M. ERK1/2 signaling regulates the immune microenvironment and macrophage recruitment in glioblastoma. Biosci Rep (2019) 39(9):BSR20191433. doi: 10.1042/BSR20191433 PMC6744584 31467175

[103] Wu JY Huang TW Hsieh YT Wang YF Yen CC Lee GL. Cancer-Derived Succinate Promotes Macrophage Polarization and Cancer Metastasis via Succinate Receptor. Mol Cell (2020) 77(2):213–227.e215. doi: 10.1016/j.molcel.2019.10.023 31735641

[104] Justus CR Dong L Yang LV. Acidic tumor microenvironment and pH-sensing G protein-coupled receptors. Front Physiol (2013) 4:354. doi: 10.3389/fphys.2013.00354 24367336 PMC3851830

[105] Mead JR Hughes TR Irvine SA Singh NN Ramji DP. Interferon-gamma stimulates the expression of the inducible cAMP early repressor in macrophages through the activation of casein kinase 2. A potentially novel pathway for interferon-gamma-mediated inhibition of gene transcription. J Biol Chem (2003) 278(20):17741–51. doi: 10.1074/jbc.M301602200 12609974

[106] Bjarnadóttir TK Gloriam DE Hellstrand SH Kristiansson H Fredriksson R Schiöth HB. Comprehensive repertoire and phylogenetic analysis of the G protein-coupled receptors in human and mouse. Genomics (2006) 88(3):263–73. doi: 10.1016/j.ygeno.2006.04.001 16753280

[107] Vadevoo SMP Gunassekaran GR Lee C Lee N Lee J Chae S. The macrophage odorant receptor Olfr78 mediates the lactate-induced M2 phenotype of tumor-associated macrophages. Proc Natl Acad Sci U.S.A. (2021) 118(37):e2102434118. doi: 10.1073/pnas.2102434118 PMC8449333 34504016

[108] Ranganathan P Shanmugam A Swafford D Suryawanshi A Bhattacharjee P Hussein MS. GPR81, a Cell-Surface Receptor for Lactate, Regulates Intestinal Homeostasis and Protects Mice from Experimental Colitis. J Immunol (2018) 200(5):1781–9. doi: 10.4049/jimmunol.1700604 PMC5858928 29386257

[109] Liu C Wu J Zhu J Kuei C Yu J Shelton J. Lactate inhibits lipolysis in fat cells through activation of an orphan G-protein-coupled receptor, GPR81. J Biol Chem (2009) 284(5):2811–22. doi: 10.1074/jbc.M806409200 19047060

[110] Brown TP Bhattacharjee P Ramachandran S Sivaprakasam S Ristic B Sikder MOF. The lactate receptor GPR81 promotes breast cancer growth via a paracrine mechanism involving antigen-presenting cells in the tumor microenvironment. Oncogene (2020) 39(16):3292–304. doi: 10.1038/s41388-020-1216-5 32071396

[111] Xie Q Zhu Z He Y Zhang Z Zhang Y Wang Y. A lactate-induced Snail/STAT3 pathway drives GPR81 expression in lung cancer cells. Biochim Biophys Acta Mol Basis Dis (2020) 1866(1):165576. doi: 10.1016/j.bbadis.2019.165576 31666207

[112] Feng J Yang H Zhang Y Wei H Zhu Z Zhu B. Tumor cell-derived lactate induces TAZ-dependent upregulation of PD-L1 through GPR81 in human lung cancer cells. Oncogene (2017) 36(42):5829–39. doi: 10.1038/onc.2017.188 28604752

[113] Qu J Sun Z Peng C Li D Yan W Xu Z. C. tropicalis promotes chemotherapy resistance in colon cancer through increasing lactate production to regulate the mismatch repair system. Int J Biol Sci (2021) 17(11):2756–69. doi: 10.7150/ijbs.59262 PMC8326116 34345205

[114] Wanders D Graff EC Judd RL. Effects of high fat diet on GPR109A and GPR81 gene expression. Biochem Biophys Res Commun (2012) 425(2):278–83. doi: 10.1016/j.bbrc.2012.07.082 22842580

[115] Jeninga EH Bugge A Nielsen R Kersten S Hamers N Dani C. Peroxisome proliferator-activated receptor gamma regulates expression of the anti-lipolytic G-protein-coupled receptor 81 (GPR81/Gpr81). J Biol Chem (2009) 284(39):26385–93. doi: 10.1074/jbc.M109.040741 PMC2785326 19633298

[116] Raychaudhuri D Bhattacharya R Sinha BP Liu CSC Ghosh AR Rahaman O. Lactate Induces Pro-tumor Reprogramming in Intratumoral Plasmacytoid Dendritic Cells. Front Immunol (2019) 10:1878. doi: 10.3389/fimmu.2019.01878 31440253 PMC6692712

[117] Hoque R Farooq A Ghani A Gorelick F Mehal WZ. Lactate reduces liver and pancreatic injury in Toll-like receptor- and inflammasome-mediated inflammation via GPR81-mediated suppression of innate immunity. Gastroenterology (2014) 146(7):1763–74. doi: 10.1053/j.gastro.2014.03.014 PMC4104305 24657625

[118] Yang K Xu J Fan M Tu F Wang X Ha T. Lactate Suppresses Macrophage Pro-Inflammatory Response to LPS Stimulation by Inhibition of YAP and NF-κB Activation via GPR81-Mediated Signaling. Front Immunol (2020) 11:587913. doi: 10.3389/fimmu.2020.587913 33123172 PMC7573489

[119] Samuvel DJ Sundararaj KP Nareika A Lopes-Virella MF Huang Y. Lactate boosts TLR4 signaling and NF-kappaB pathway-mediated gene transcription in macrophages via monocarboxylate transporters and MD-2 up-regulation. J Immunol (2009) 182(4):2476–84. doi: 10.4049/jimmunol.0802059 PMC2673542 19201903

[120] Hanahan D Weinberg RA. Hallmarks of cancer: the next generation. Cell (2011) 144(5):646–74. doi: 10.1016/j.cell.2011.02.013 21376230

[121] DeBerardinis RJ Lum JJ Hatzivassiliou G Thompson CB. The biology of cancer: metabolic reprogramming fuels cell growth and proliferation. Cell Metab (2008) 7(1):11–20. doi: 10.1016/j.cmet.2007.10.002 18177721

[122] Martínez-Reyes I Chandel NS. Waste Not, Want Not: Lactate Oxidation Fuels the TCA Cycle. Cell Metab (2017) 26(6):803–4. doi: 10.1016/j.cmet.2017.11.005 29211977

[123] Cassim S Raymond VA Dehbidi-Assadzadeh L Lapierre P Bilodeau M. Metabolic reprogramming enables hepatocarcinoma cells to efficiently adapt and survive to a nutrient-restricted microenvironment. Cell Cycle (2018) 17(7):903–16. doi: 10.1080/15384101.2018.1460023 PMC6056217 29633904

[124] Hui S Ghergurovich JM Morscher RJ Jang C Teng X Lu W. Glucose feeds the TCA cycle via circulating lactate. Nature (2017) 551(7678):115–8. doi: 10.1038/nature24057 PMC5898814 29045397

[125] Tasdogan A Faubert B Ramesh V Ubellacker JM Shen B Solmonson A. Metabolic heterogeneity confers differences in melanoma metastatic potential. Nature (2020) 577(7788):115–20. doi: 10.1038/s41586-019-1847-2 PMC6930341 31853067

[126] Dart A. Tumour metabolism: Lactic acid: not just a waste product? Nat Rev Cancer (2016) 16(11):676–7. doi: 10.1038/nrc.2016.109 27713543

[127] Ippolito L Morandi A Giannoni E Chiarugi P. Lactate: A Metabolic Driver in the Tumour Landscape. Trends Biochem Sci (2019) 44(2):153–66. doi: 10.1016/j.tibs.2018.10.011 30473428

[128] Cassim S Pouyssegur J. Tumor Microenvironment: A Metabolic Player that Shapes the Immune Response. Int J Mol Sci (2019) 21(1):157. doi: 10.3390/ijms21010157 31881671 PMC6982275

[129] Brand A Singer K Koehl GE Kolitzus M Schoenhammer G Thiel A. LDHA-Associated Lactic Acid Production Blunts Tumor Immunosurveillance by T and NK Cells. Cell Metab (2016) 24(5):657–71. doi: 10.1016/j.cmet.2016.08.011 27641098

[130] Renner K Bruss C Schnell A Koehl G Becker HM Fante M. Restricting Glycolysis Preserves T Cell Effector Functions and Augments Checkpoint Therapy. Cell Rep (2019) 29(1):135–150.e139. doi: 10.1016/j.celrep.2019.08.068 31577944

[131] Najafi M Hashemi Goradel N Farhood B Salehi E Nashtaei MS Khanlarkhani N. Macrophage polarity in cancer: A review. J Cell Biochem (2019) 120(3):2756–65. doi: 10.1002/jcb.27646 30270458

[132] Pascual-García M Bonfill-Teixidor E Planas-Rigol E Rubio-Perez C Iurlaro R Arias A. LIF regulates CXCL9 in tumor-associated macrophages and prevents CD8(+) T cell tumor-infiltration impairing anti-PD1 therapy. Nat Commun (2019) 10(1):2416. doi: 10.1038/s41467-019-10369-9 31186412 PMC6559950

[133] Zhang L Li S. Lactic acid promotes macrophage polarization through MCT-HIF1α signaling in gastric cancer. Exp Cell Res (2020) 388(2):111846. doi: 10.1016/j.yexcr.2020.111846 31945319

[134] Xu JY Wang WS Zhou J Liu CY Shi JL Lu PH. The Importance of a Conjoint Analysis of Tumor-Associated Macrophages and Immune Checkpoints in Pancreatic Cancer. Pancreas (2019) 48(7):904–12. doi: 10.1097/MPA.0000000000001364 31268976

[135] Shan T Chen S Chen X Wu T Yang Y Li S. M2−TAM subsets altered by lactic acid promote T−cell apoptosis through the PD−L1/PD−1 pathway. Oncol Rep (2020) 44(5):1885–94. doi: 10.3892/or.2020.7767 PMC7551099 33000216

[136] Papadaki C Manolakou S Lagoudaki E Pontikakis S Ierodiakonou D Vogiatzoglou K. Correlation of PKM2 and CD44 Protein Expression with Poor Prognosis in Platinum-Treated Epithelial Ovarian Cancer: A Retrospective Study. Cancers (Basel) (2020) 12(4):1013. doi: 10.3390/cancers12041013 PMC7225941 32326107

[137] Li H Yan M Wu X Wang Y Huang L. Expression and clinical significance of pyruvate kinase M2 in breast cancer: A protocol for meta-analysis and bioinformatics validation analysis. Med (Baltimore) (2021) 100(18):e25545. doi: 10.1097/MD.0000000000025545 PMC8104244 33950928

[138] Sfakianaki M Papadaki C Tzardi M Trypaki M Manolakou S Messaritakis I. PKM2 Expression as Biomarker for Resistance to Oxaliplatin-Based Chemotherapy in Colorectal Cancer. Cancers (Basel) (2020) 12(8):2058. doi: 10.3390/cancers12082058 PMC7465271 32722474

[139] Chen J Xie J Jiang Z Wang B Wang Y Hu X. Shikonin and its analogs inhibit cancer cell glycolysis by targeting tumor pyruvate kinase-M2. Oncogene (2011) 30(42):4297–306. doi: 10.1038/onc.2011.137 21516121

[140] Li W Liu J Jackson K Shi R Zhao Y. Sensitizing the therapeutic efficacy of taxol with shikonin in human breast cancer cells. PloS One (2014) 9(4):e94079. doi: 10.1371/journal.pone.0094079 24710512 PMC3977981

[141] Liu T Li S Wu L Yu Q Li J Feng J. Experimental Study of Hepatocellular Carcinoma Treatment by Shikonin Through Regulating PKM2. J Hepatocell Carcinoma (2020) 7:19–31. doi: 10.2147/JHC.S237614 32110554 PMC7035901

[142] Wang H Tang Y Fang Y Zhang M Wang H He Z. Reprogramming Tumor Immune Microenvironment (TIME) and Metabolism via Biomimetic Targeting Codelivery of Shikonin/JQ1. Nano Lett (2019) 19(5):2935–44. doi: 10.1021/acs.nanolett.9b00021 30950276

[143] Li J Zhao M Sun M Wu S Zhang H Dai Y. Multifunctional Nanoparticles Boost Cancer Immunotherapy Based on Modulating the Immunosuppressive Tumor Microenvironment. ACS Appl Mater Interfaces (2020) 12(45):50734–47. doi: 10.1021/acsami.0c14909 33124808

[144] Mossmann D Park S Hall MN. mTOR signalling and cellular metabolism are mutual determinants in cancer. Nat Rev Cancer (2018) 18(12):744–57. doi: 10.1038/s41568-018-0074-8 30425336

[145] Deng L Qian G Zhang S Zheng H Fan S Lesinski GB. Inhibition of mTOR complex 1/p70 S6 kinase signaling elevates PD-L1 levels in human cancer cells through enhancing protein stabilization accompanied with enhanced β-TrCP degradation. Oncogene (2019) 38(35):6270–82. doi: 10.1038/s41388-019-0877-4 31316145

[146] Chen B Gao A Tu B Wang Y Yu X Wang Y. Metabolic modulation via mTOR pathway and anti-angiogenesis remodels tumor microenvironment using PD-L1-targeting codelivery. Biomaterials (2020) 255:120187. doi: 10.1016/j.biomaterials.2020.120187 32590192

[147] Crane C Panner A Pieper RO Arbiser J Parsa AT. Honokiol-mediated inhibition of PI3K/mTOR pathway: a potential strategy to overcome immunoresistance in glioma, breast, and prostate carcinoma without impacting T cell function. J Immunother (2009) 32(6):585–92. doi: 10.1097/CJI.0b013e3181a8efe6 PMC3795513 19483651

[148] Zheng Z Zhang J Jiang J He Y Zhang W Mo X. Remodeling tumor immune microenvironment (TIME) for glioma therapy using multi-targeting liposomal codelivery. J Immunother Cancer (2020) 8(2):e000207. doi: 10.1136/jitc-2019-000207 PMC7437977 32817393

[149] Zhang Y Liu C Wu C Song L. Natural peptides for immunological regulation in cancer therapy: Mechanism, facts and perspectives. BioMed Pharmacother (2023) 159:114257. doi: 10.1016/j.biopha.2023.114257 36689836

[150] Ciscato F Ferrone L Masgras I Laquatra C Rasola A. Hexokinase 2 in Cancer: A Prima Donna Playing Multiple Characters. Int J Mol Sci (2021) 22(9):4716. doi: 10.3390/ijms22094716 PMC8125560 33946854

[151] Zhang SQ Yung KK Chung SK Chung SS. Aldo-keto reductases-mediated cytotoxicity of 2-deoxyglucose: A novel anticancer mechanism. Cancer Sci (2018) 109(6):1970–80. doi: 10.1111/cas.13604 PMC5989857 29617059

[152] Minor RK Smith DL Jr. Sossong AM Kaushik S Poosala S Spangler EL. Chronic ingestion of 2-deoxy-D-glucose induces cardiac vacuolization and increases mortality in rats. Toxicol Appl Pharmacol (2010) 243(3):332–9. doi: 10.1016/j.taap.2009.11.025 PMC2830378 20026095

[153] Sasaki K Nishina S Yamauchi A Fukuda K Hara Y Yamamura M. Nanoparticle-Mediated Delivery of 2-Deoxy-D-Glucose Induces Antitumor Immunity and Cytotoxicity in Liver Tumors in Mice. Cell Mol Gastroenterol Hepatol (2021) 11(3):739–62. doi: 10.1016/j.jcmgh.2020.10.010 PMC7841526 33191170

[154] Andrzejewski S Siegel PM St-Pierre J. Metabolic Profiles Associated With Metformin Efficacy in Cancer. Front Endocrinol (Lausanne) (2018) 9:372. doi: 10.3389/fendo.2018.00372 30186229 PMC6110930

[155] Salani B Del Rio A Marini C Sambuceti G Cordera R Maggi D. Metformin, cancer and glucose metabolism. Endocr Relat Cancer (2014) 21(6):R461–471. doi: 10.1530/ERC-14-0284 25273809

[156] Elgogary A Xu Q Poore B Alt J Zimmermann SC Zhao L. Combination therapy with BPTES nanoparticles and metformin targets the metabolic heterogeneity of pancreatic cancer. Proc Natl Acad Sci U.S.A. (2016) 113(36):E5328–5336. doi: 10.1073/pnas.1611406113 PMC5018752 27559084

[157] Hogg SJ Vervoort SJ Deswal S Ott CJ Li J Cluse LA. BET-Bromodomain Inhibitors Engage the Host Immune System and Regulate Expression of the Immune Checkpoint Ligand PD-L1. Cell Rep (2017) 18(9):2162–74. doi: 10.1016/j.celrep.2017.02.011 PMC5340981 28249162

[158] Zhu H Bengsch F Svoronos N Rutkowski MR Bitler BG Allegrezza MJ. BET Bromodomain Inhibition Promotes Anti-tumor Immunity by Suppressing PD-L1 Expression. Cell Rep (2016) 16(11):2829–37. doi: 10.1016/j.celrep.2016.08.032 PMC5177024 27626654

[159] He Y Fang Y Zhang M Zhao Y Tu B Shi M. Remodeling "cold" tumor immune microenvironment via epigenetic-based therapy using targeted liposomes with in *situ* formed albumin corona. Acta Pharm Sin B (2022) 12(4):2057–73. doi: 10.1016/j.apsb.2021.09.022 PMC9279642 35847495

[160] Fan T Sun G Sun X Zhao L Zhong R Peng Y. Tumor Energy Metabolism and Potential of 3-Bromopyruvate as an Inhibitor of Aerobic Glycolysis: Implications in Tumor Treatment. Cancers (Basel) (2019) 11(3):317. doi: 10.3390/cancers11030317 PMC6468516 30845728

[161] El Sayed SM Abou El-Magd RM Shishido Y Chung SP Sakai T Watanabe H. D-amino acid oxidase gene therapy sensitizes glioma cells to the antiglycolytic effect of 3-bromopyruvate. Cancer Gene Ther (2012) 19(1):1–18. doi: 10.1038/cgt.2011.59 21921941

[162] Zhang Y Wei J Xu J Leong WS Liu G Ji T. Suppression of Tumor Energy Supply by Liposomal Nanoparticle-Mediated Inhibition of Aerobic Glycolysis. ACS Appl Mater Interfaces (2018) 10(3):2347–53. doi: 10.1021/acsami.7b16685 29286239

[163] Dichtl S Lindenthal L Zeitler L Behnke K Schlösser D Strobl B. Lactate and IL6 define separable paths of inflammatory metabolic adaptation. Sci Adv (2021) 7(26):eabg3505. doi: 10.1126/sciadv.abg3505 PMC8221612 34162546

